# Enlarged vestibular aqueduct syndrome: report of 3 cases and literature review

**DOI:** 10.1016/S1808-8694(15)31342-2

**Published:** 2015-10-20

**Authors:** José A. Pinto, Carlos Fernando Mello, Ana Carla S. Marqui, Delmer J. Perfeito, Roberto D.P. Ferreira, Rubens H. Silva

**Affiliations:** ^1^ Director, Nucleus of Otorhinolaryngology and Head and Neck Surgery, Sao Paulo; ^2^ Radiologist, Hospital e Maternidade São Camilo – SP; ^3^ Resident Physician, Nucleus of Otorhinolaryngology and Head and Neck Surgery, Sao Paulo

**Keywords:** enlarged vestibular aqueduct, floating sensorineural hearing loss, mixed hearing loss, child

## Abstract

**E**nlarged Vestibular Aqueduct Syndrome is characterized by a widening of the vestibular aqueduct, associated with sensorineural hearing loss, or sometimes with mixed hearing loss, which may be congenital or acquired during childhood. The sensorineural hearing loss may be classified into mild, moderate and severe, associated with sudden periods of improvement or aggravation. The enlargement of the vestibular aqueduct is the most common inner ear anomaly. This syndrome is admitted as a result of a genetic abnormality of the vestibular aqueduct development, previous to the fifth week of gestation. The incidence of this syndrome ranges from 1% to 1.3%, with the possibility of getting up to 7%, depending on the examined population. The aim of this study was to analyze three cases of LVAS seen at the Otorhinolaryngology and Radiology Department of Sao Camilo Hospital - Sao Paulo. Two of these three cases were of brothers, from the same mother but from different fathers. Two were male and one was female and the ages ranged from 9 to 30 years old. The diagnostic method of election was CT - Computerized Tomography of the temporal bones. The recommended management of the cases was conservative, except for those of cranial trauma, barotrauma and, if necessary, the use of hearing aids.

## INTRODUCTION

Large Vestibular Aqueduct (LVA) is known as the most common form of inner ear anomaly and it may be radiologically diagnosed using temporal bone computed tomography (CT scan) and inner ear Magnetic Resonance Imaging (MRI). Non-syndromic sensorineural hearing loss (NSSHL) associated with LVA has been a source of interest because it is associated with future clinical characteristics, including hearing fluctuation, many cases of progressive sensorineural hearing loss, sometimes mixed loss, which normally starts in childhood and may also be associated with vestibular symptoms. Large Vestibular Aqueduct Syndrome (LVAS) was described for the first time by Valvassori and Clemis^1^ in 1978 in a retrospective study with 3,700 inner ear CT scans, in which they reported findings of 50 patients with LVA, more frequently found in bilateral cases. Forty percent of LVAS cases were isolated anomalies, whereas 60% of them had some associated abnormality, normally enlargement of lateral semicircular canal. LVAS may be associated with Mondini's dysplasia and more recently, with Pendred Syndrome. Swartze et al.^2^ have also described 3 cases of sensorineural hearing loss related with LVA and confirmed by CT scan, in which aqueduct enlargement was on the same side as the hearing loss.

In children, differential diagnosis of sensorineural loss should be made with congenital infectious diseases (cytomegalovirus, syphilis, intrauterine infections, and less frequently, rubella and toxoplasmosis) or acquired diseases (bacterial meningitis). Differential diagnosis should also be made with HIV infection because it is a neurotrophic virus. Moreover, other causes should be investigated, such as ototoxicity, autoimmune diseases, traumatic, vascular and hereditary diseases. Even after investigating such factors, the etiology may remain unknown. Bento et al.^3^, in 2001, used the following protocol in the investigation of possible etiopathogenic factors involved in the origin of the disease, which are: complete blood count, hemosedimentation rate, glycemic curve, and 5-hour insulin levels, serological tests for syphilis, toxoplasmosis, cytomegalovirus, rubella, measles, autoimmune disease factors (rheumatoid factor, C3, C4, reactive C protein, LE cell, antinucleus factor, ASLO, native anti-DNA, alpha 1 glucoprotein, and IgE dosage), which were all used in our protocol.

## CASE REPORT

### Case 1

T.R.M.P, female, 30 years of age, born and living in Osasco–SP, with history of intermittent rotation dizziness and tinnitus for 6 months. She was medicated with Gingko Biloba (80 mg BID) and ordered vectoelectronystagmography with diagnosis of Irritative Peripheral Vestibular Syndrome. She presented tinnitus, disabling dizziness and retromastoid pain on the left. She also reported progressive hearing loss of fluctuating characteristics since childhood (bilateral sensorineural hearing loss detected at audiometry), of undefined cause, using a hearing aid on the left since the age of 8 years. In her clinical history she presented fetal distress at birth and language development delay in childhood. ENT examination revealed at otoscopy tympanic membrane opacity and no other findings. We ordered temporal bone CT scan, pure tone audiometry and vocal discrimination, immittanciometry, ABR, head self-rotation, complete blood test, fast glucose, serological tests for syphilis, toxoplasmosis, cytomegalovirus, rubella and measles; autoimmune disease factors (rheumatoid factor, C3, C4, reactive C protein, LE cells, antinucleus factor, ASLO, native anti-DNA, alpha 1 glucoprotein, hemosedimentation rate and IgE dosage).

**Figure 1:**
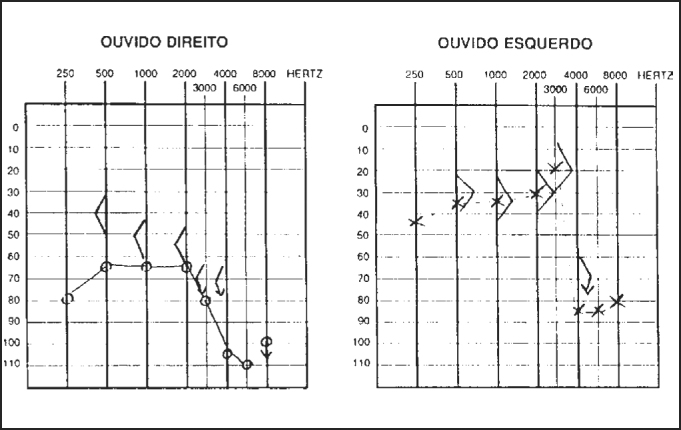
Audiometry with sensorineural hearing loss on the left and mixed loss on the right.

**Figure 2:**
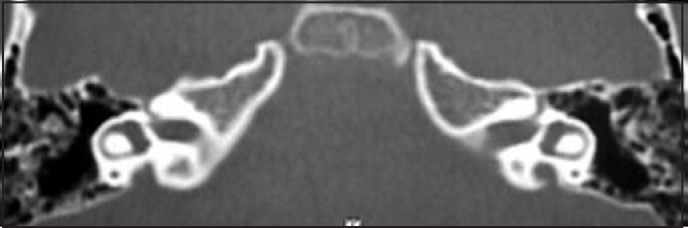
CT scan showing bilateral large vestibular aqueduct.

Pure tone audiometry and vocal discrimination showed moderate to severe mixed loss in speech frequencies and profound loss in high frequencies on the right and mild sensorineural loss in speech frequencies and severe loss in high frequencies on the left (Figure 1). Brainstem audiometry demonstrated absence of responses at 90dB on the right and 85dB on the left. Head auto-rotation showed dysfunction of the vestibular aqueduct. Temporal bone CT scan showed dilated aspect of distal segment of vestibular aqueducts on the region of the endolymphatic sacs (Figure 2). Normal serological tests, IgE of 564 UI/ml (nl = 87 UI/ml), fast glucose of 54 mg/dl and negative autoimmune disease factors except for antinucleus factor, which was positive (1/40).

### Case 2

**Figure 3:**
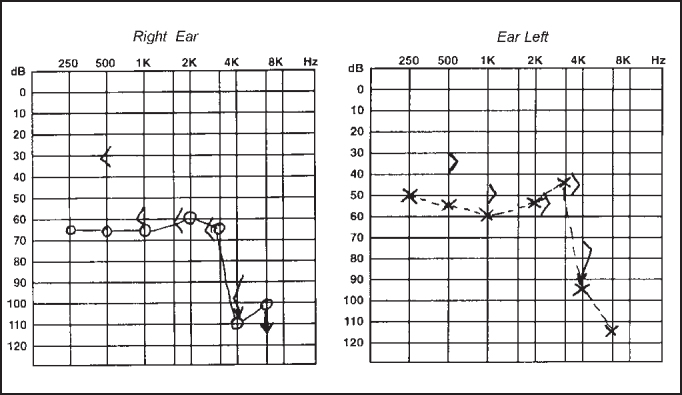
CT scan showing bilateral large vestibular aqueduct, especially on the right.

**Figure 4:**
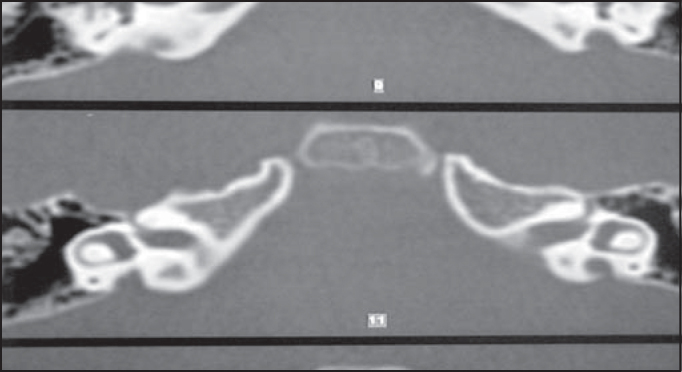
Audiometry showing bilateral mixed hearing loss.

R.R.R.S.G, male, 9 years, born and living in Sao Paulo, with history of progressive and fluctuating bilateral hearing loss since the age of 2 years, especially on the right, without apparent cause. According to the mother, the child spoke too loud and needed the TV volume always up. He did not report otalgia or otorrhea. The patient reported episodes of constant tinnitus, such as a whistle, especially on the left. Did not report dizziness. Uses bilateral hearing aids since the age of 7 years. He presented history of deafness in the family (sister, cousins, uncles and grandfather's brother). He is the only child of the mother's second marriage. Gestation and delivery were uneventful. The physical examination presented normal otoscopy and oroscopy, and rhinoscopy showed pale and hypertrophic lower conchae. Audiometry showed moderate to severe mixed loss on the right and moderate loss on the left (Figure 3). Temporal bone CT scan showed enlargement of vestibular aqueducts, especially on the right, with normal aspect of cochlea and vestibule (Figure 4).

### Case 3

**Figure 5:**
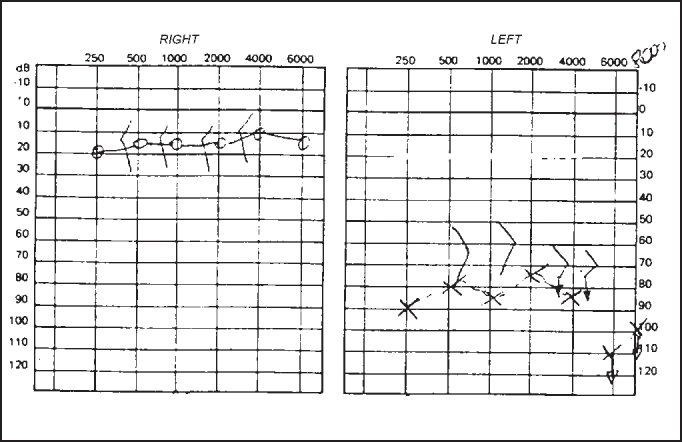
Audiometry showing mixed hearing loss on the left.

**Figure 6:**
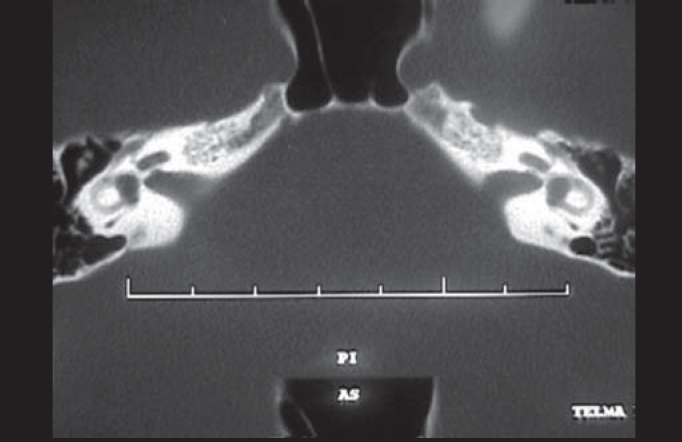
CT showing enlargement of left vestibular aqueduct.

B.R.G, female, 16 years, born and living in Sao Paulo. The mother reported that 10 years before she detected hearing loss on the left when she realized the daughter did not respond to sounds coming from sources on the left side. She looked for an ENT doctor, who asked for audiometry and confirmed severe hearing loss on the left without any apparent defined cause. For the past 4 years she has had constant mild rotation dizziness, with no other associated symptoms. She has not worn hearing aids. History of deafness in the family (brother, cousin, grandmother's brother, everyone in the mother's family). Uneventful gestation and birth. As to common childhood diseases, she reported varicella at the age of 2 years. ENT examination was normal. Pure tone audiometry presented severe mixed hearing loss on the left (Figure 5). Temporal bone CT scan evidenced large aqueduct on the left with normal cochlea and vestibule (Figure 6).

The main symptoms of the patient are summed up in Table 2.

**Table 2:** Clinical case of patients with the main signs and symptoms of LVAS.

	Gender	Age	Type of hearing loss	Vertigo	tinnitus
CASE 1	Female	30 years	RE: moderate severe mixedLE: mild sensorineural	Present	Present
CASE 2	Male	9 years	RE: moderate severe mixedLE: moderate mixed	Absent	Present
CASE 3	Female	16 years	RE: NormalLE: severe mixed	Present	Absent

## DISCUSSION

Vestibular aqueduct (VA) is a bony canal that communicates the posterior-medial portion of the epidural space vestibule with the medium posterior fossa (Zazal, 1995)^4^. Endolymphatic duct runs inside the VA connecting the endolymphatic sac and the vestibule labyrinth, and through the reuniens duct it gets together with the cochlear duct.

Whereas the other portions of the ear have been developed since birth, vestibular aqueduct and endolymphatic sac are immature and small. As the posterior cranial fossa is expanded, VA and endolymphatic sac quickly increase in size and reach maturity at about the age of 4 years

Using graphic reconstruction techniques, Kodama and Sando^5^ concluded that there is a direct and proportional correlation between VA area and volume, that is, if there is VA enlargement, there is also enlargement at least of the rugous portion of the endolymphatic sac. In turn, it may contribute to an active ionic exchange of endolymph with cerebrospinal fluid and also serve as a reservoir of endolymph in addition to having a regulating role in pressure because it is capable of absorbing water.

VA is considered normal when it does not exceed the diameter of the adjacent semicircular canal or when it is inferior to 1.5mm.

LVAS is characterized by enlargement associated with, which may also be mixed, with no defined cause. One of the many hypotheses considered are affection to homeostasis in endolymphatic circulation as a result of ductal enlargement, with consequent damage to cochlear neuroepithelium^6^. Another hypothesis is whether enlargement of VA and endolymphatic sac would not be an isolated anomaly, but rather a continuity of the cochleovestibular malformation. Lemmerling and Antonelli^7^ reported an association of defects in the modiolus of patients with LVA.

Antonelli classified VA enlargement into 5 grades. This measure is made from the intermediate point between the external opening and the common crura region.

- GRADE I: lumen of the aqueduct is only visualized in the temporal bone cortex.
- GRADE II: lumen of the aqueduct is visible close to common crura.
- GRADE III: lumen of the aqueduct is larger than the common crura, but it is not visible in the topography of the vestibule output.
- GRADE IV: the internal portion of the aqueduct is visible and its diameter in the output topography is smaller or equal to the common crura.
- GRADE V: the internal portion of the aqueduct is visible and the diameter of the output topography is larger than the diameter of the common crura.

Initially we thought that the hearing loss would remain unaltered during the whole life. Later, it was noticed that the hearing loss could fluctuate or deteriorate progressively^8^, which may sometimes start from head traumas^9^.

Children with LVAS may have moderate or severe hearing loss in childhood, but residual hearing allows the child to get adapted to some situations, such as for example, to go to school and to develop spoken language with the use of conventional hearing aids^10^. Many of these children need support, such as sign language for communication.

Recently, much attention has been given to the hereditary nature of this syndrome. Griffth et al.^11^ described a family case of hearing loss associated with EVA. Abe and Ussami^12^ have also described 6 cases in 3 families with hearing loss associated with EVA. Ussami et al.^13^ reported the location of the gene responsible for sensorineural hearing loss associated with EVA, which is located in the chromosome region 7q31, whose genetic chromosome characteristic is recessive autosomal. This region was also described as being the gene responsible for Pendred Syndrome, which is characterized by the association between hearing loss, thyroid goiter, and positive perchloride test. These genetic affection may also be related with Mondini's dysplasia.

A reported possibility is that the gene of sensorineural hearing loss is located at the same gene of Pendred syndrome. The most widely accepted hypothesis is that a gene mutation would cause NSSHL in patients with LVAS, which makes us believe that different mutations to the Pendred gene would cause different phenotypes and also non-syndromic sensorineural hearing loss associated with LVA and Pendred syndrome. According to Urman and Talbot^14^ if LVA is found isolated, then its presence is owed to a genetic mutation of Pendred syndrome.

Clinical picture of LVAS is variable, normally its onset is at childhood and there is moderate to severe fluctuating or progressive loss; sometimes, it may also be sudden. Vertigo may be present and it is more common than it is believed.

There is no audiometric curve that characterizes or indicates the hypothesis of LVAS, reason why we should be attentive in cases of mixed loss and NSSHL in childhood. Abe et al.^12^ reported that one of the main characteristics of LVAS is fluctuating NSSHL, especially in high frequencies.

Current studies (Zazal, 1995)^4^ demonstrated that there is no correlation at the level of hearing loss with age or width of aqueduct. We have also observed the presence of endolymphatic system at MRI, which has proven to be better than CT scan for the study of LVAS, especially for the visualization of dilation of endolymphatic duct and sac and also other malformations of the inner ear and cochlear hypoplasia.

In our case reports, patients 2 and 3 were brothers by mother, with respective ages of 9 and 16 years with hearing loss since childhood, and they also presented family history of deafness (uncles and maternal grandmother). In cases 1 and 2, patients presented mixed or sensorineural loss since childhood, and tinnitus in the affected ears since childhood, which spoke in favor of LVAS and CT scan showing large vestibular aqueduct (Table 1). In the literature, there are rare reports of tinnitus and dizziness as manifestations, reported by all our patients, even though vestibular tests were normal. We also investigated congenital symptoms such as Pendred, Mondini's Dysplasia and Klippel Feil, but results were negative.

**Table 1:** Values of the vestibular aqueduct of patients.

	Right vestibular aqueduct (mm)	Left vestibular aqueduct (mm)
CASE 1	**3 mm**	**4 mm**
CASE 2	**7 mm**	**5 mm**
CASE 3	———	**4 mm**

As to LVA management, many treatment options have been described. Jackler and De La Cruz^9^ made endolymphatic shunts in 7 patients with LVA and results were negative in 4 cases, concluding that this type of intervention is contraindicated in these patients. Wilson et al.^15^ reported a technique of endolymphatic sac occlusion via intraluminar access in 7 patients, with negative results in 6 of them. Welling et al.^16^ reported the same technique via extraluminar approach in 10 patients with bilateral LVA, but there was no hearing satisfactory gain and no significant difference in audiometric results using these techniques; quite to the contrary, the resulting hearing loss made authors quit the surgical procedure.

In the reported cases, treatment has been conservative: to prevent head trauma, barotrauma and radical sports to try to reduce the risk of hearing loss by trauma. Patients 1 and 2 had used hearing aids respectively for 22 and 2 years with hearing improvement. Patient 3 did not use hearing aid because he reported he could hear well and did not need hearing aids. Patient 1 is not taking anti-vertigo medication, because he has been following a diet after the detection of hypoglycemia, which may be a possible cause of dizziness. Relatives of cases 2 and 3 are currently being studied because there were hearing losses to be defined.

As to cochlear implant, recent studies have confirmed that it serves to maintain sufficient hearing to integrate the children in regular schools^17^. Aschendorff et al.^10^ reported that children with cochlear implant and LVAS would have results similar to other implanted patients with normal inner ears. The difference between LVAS and congenital deafness is that children with LVAS acquire speech and language before children who have profound loss. Au and Gibson^17^ reported cochlear implant use in 10 school-aged children whose mean age was 6 to 8 years (ranging from 2 years and 3 months to 9 years and 10 months) with audiometry that showed severe hearing loss. After cochlear implant, they performed postoperative audiometry (6, 12 months and 2 years) that demonstrated significant hearing improvement in these children. The authors concluded that cochlear implants are an effective resource in managing hearing loss in children owing to deterioration of cochlear function with LVA.

However, there are no comparative studies showing hearing gain in LVAS implanted patients after the age of 9 years, which made us maintain our conservative management approach with these patients.

## CONCLUSION

LVAS is a clinical entity that should be part of the differential diagnosis of sensorineural hearing loss, progressive mixed loss genetic syndromes in children. Early diagnosis is essential because it allows more favorable rehabilitation treatment such as cochlear implant, which has shown favorable results when the diagnosis of LVAS is made at an early age.
